# Unpacking the Itch Score: A Critical Examination of Routine Itch Measurement in Dermatology Practice

**DOI:** 10.1016/j.xjidi.2025.100351

**Published:** 2025-01-21

**Authors:** Serene Majid, Steven R. Feldman

**Affiliations:** 1 Center for Dermatology Research, Department of Dermatology, Wake Forest University School of Medicine, Winston-Salem, North Carolina, USA; 2 Department of Dermatology, Wake Forest University School of Medicine, Winston-Salem, North Carolina, USA; 3 Department of Pathology, Wake Forest University School of Medicine, Winston-Salem, North Carolina, USA; 4 Department of Social Sciences & Health Policy, Wake Forest University School of Medicine, Winston-Salem, North Carolina, USA

**Keywords:** Dermatology, Pruritus, Health disparities, Itch measurement tools, Itch score

## Summary of Key Findings

The article “Capture of Patient Itch Scores in Practice Reveals Disparate Itch Impact Based on Age, Gender, and Race: A Cross-Sectional Survey Analysis” (Li and Swerlick, 2025) explores the impact of itch on health-related QOL (HRQOL) across a diverse cohort of 4892 dermatology patients. The study aims to better understand patient itch burdens and the associated HRQOL in addition to exploring the feasibility of using validated itch measurement tools (ItchyQuant and Skindex-Mini) in routine clinical practice to assess itch severity and its relationship to demographic factors such as age, gender, and race. Itch severity varied, with African American patients, females, and older patients reporting higher itch burdens than other groups. Further research on the causes of these disparities may be valuable. The investigators conclude, we believe prematurely, that, “routine itch assessments should be implemented in dermatologic practice.”

**“**To support the assertion that validated itch measures *should* be used, randomized controlled trials comparing the outcomes of patients who have their itch scores captured with those who receive the current standard of care are needed.”

## Strengths and Clinical Relevance

The use of validated tools such as ItchyQuant and Skindex-Mini in this study is consistent with the evolving focus of dermatology research on multidimensional assessments of itch. These approaches capture itch intensity, address its impact on QOL, and are increasingly used in clinical research (Erickson and Kim, 2019). The inclusion of 4892 patients from diverse demographic backgrounds allows for capturing the variable nature and impact of pruritus among distinct demographic groups, thereby providing new insights that may be investigated in larger multicenter trials. Integrating routine patient questionnaires into dermatology practice was feasible and may aid clinicians in recognizing the multifactorial nature of their patients’ pruritus. Moreover, with a survey engagement rate of 50.7%, the questionnaire approach may be used in further studies to provide a more comprehensive view of patient health beyond visible skin lesions.

The racial disparity in itch severity was striking, particularly the higher itch burden in African Americans, females, and older patients. Further research on the underlying causes of these disparities, which could include genetic factors, differences in skin conditions, and variations in access to care, is important. Broader healthcare disparities may exist in dermatology, where certain conditions may be underrecognized in darker skin tones. Understanding the full scope of a patient’s health requires not only addressing the visible symptoms but also considering how social, racial, and economic factors may influence the severity and management of dermatologic conditions. Addressing disparities can improve patient health and ensure high-quality treatment for all populations (McColl et al, 2021; Narla et al, 2023).

Pruritus can be assessed using objective and subjective methods. Although objective measures, such as monitoring scratching behavior, provide valuable insights, their connection to QOL is not entirely direct. Although excoriation is often appreciated in the setting of atopic dermatitis (AD), patients with idiopathic itch may lack visible lesions despite reporting higher itch severity. Conversely, excoriation may occur in the absence of itching (Erickson and Kim, 2019). Incorporating subjective metrics helps capture the patient’s experience more fully. The inclusion of both subjective and objective assessments contributes to the understanding and capturing of the multifaceted impact of pruritus and its differential impact on distinct demographic groups.

## Limitations

Although Li and Swerlick (2025)’s study is meaningful in its capture of a robust set of data to examine itch severity and its association with HRQOL in patients with dermatology, it has several limitations. First, the authors claim that clinicians are unlikely to address the subjective impact of symptoms such as itching unless they are made aware of it. However, this may or may not have been the case. For example, in treating conditions such as psoriasis and AD, relief from pruritus is often achieved with the resolution of underlying skin lesions, suggesting that treatment efforts can prioritize disease remission rather than focusing on itching as an isolated symptom (Szepietowski and Reich, 2016). In addition, patients with psoriasis often report a range of sensations, including stinging, pinching, tickling, and burning, which can vary significantly depending on the stage of lesion progression (Amatya et al, 2008; Zamirska et al, 2008). Nuanced descriptions of itch such as these are difficult to capture with a single standardized tool, which may fail to address the full scope of patients' experiences and inadvertently place too much emphasis on the itch dimension of the patient experience. Simply asking patients how they are doing may give clinicians the necessary information on the subjective impact of symptoms and may also capture important subjective information that a validated tool might miss.

Second, the study’s hypothesis, as stated in the introduction, revolves around the feasibility of using a standardized tool to capture itch data in routine clinical practice. However, much of the discussion strays from this central focus and delves into broader speculative claims regarding the potential benefits of measuring itch and its impact on clinical outcomes. These discussions, although interesting, are not directly tested in the study and are not adequately supported by an assessment of feasibility. To support the assertion that validated itch measures should be used, randomized controlled trials comparing the outcomes of patients who have their itch scores captured with the outcome of those who receive the current standard of care would be needed, along with cost-effectiveness data showing that the benefit would justify the cost.

## Implications for Practice

Employing a universal itch score as part of dermatological evaluation could have unintended negative consequences. The addition of another layer of paperwork increases the administrative burden on both patients and providers. The time spent completing forms can delay appointments or decrease valuable face-to-face interactions with clinicians, potentially reducing overall patient satisfaction and confidence in a physician’s capabilities (Bleustein et al, 2014). In addition, implementing a universal itch score ties into broader challenges related to patient adherence and engagement. Increasing the patient burden through additional requirements, such as universal itch assessments, may inadvertently contribute to treatment fatigue and reduced compliance (Heckman et al, 2015). Behavioral interventions that are overly frequent, effort intensive, or misaligned with patient priorities risk disengagement because adherence is closely tied to balancing patient workload and capacity. A universal itch score collected at each visit could overburden patients who already face demands for managing their health and daily lives, particularly if the form is irrelevant or unnecessary (Heckman et al, 2015).

Furthermore, whereas structured and objective measures, such as a universal itch score, may appeal to some patients, others may perceive this as an impersonal tool that distracts them from meaningful time with their provider. Patients tend to value direct communication with their physician and favor conversational interactions (Anderson et al, 2007) because these exchanges allow for rapport and trust to develop naturally between the patient and provider and foster a comfortable environment for patients to discuss concerns.

Another unintended consequence may arise if the use of quantitative scores is tied to the quality of care or provider reimbursement. If providers are judged or reimbursed on the basis of improvements in itch scores, they may be reluctant to treat individuals with refractory or challenging conditions, potentially leaving the most vulnerable patients underserved. The use of such a metric may also divert resources away from patient care, especially in resource-constrained areas, where the costs of measurement outweigh the potential benefits (Edwards, 2019).

## Suggestions for Future Research

Regular itch assessments are feasible and identify disparities. Further population-based studies are needed to investigate the factors that contribute to racial- and age-related disparities in itch severity. These studies could examine socioeconomic status, access to care, and comorbidities that might exacerbate pruritus. Interventions targeting demographics should be explored to determine whether personalized approaches to itch management would result in better patient outcomes. Future research could also explore whether integrating itch data into routine practice improves treatment outcomes and is cost-effective in the context of chronic skin conditions (Müller et al, 2023).

## ORCIDs

Serene Majid: http://orcid.org/0009-0000-1400-1985

Steven R. Feldman: http://orcid.org/0000-0002-0090-6289

## Conflict of Interest

SRF has received research, speaking, and/or consulting support from a variety of companies, including Galderma, GSK/Stiefel, Almirall, Leo Pharma, Boehringer Ingelheim, Mylan, Celgene, Pfizer, Valeant, Abbvie, Samsung, Janssen, Eli Lilly, Menlo, Merck, Novartis, Regeneron, Sanofi, Novan, Qurient, National Biological Corporation, Caremark, Advanced Medical, Sun Pharma, Suncare Research, Informa, UpToDate, and National Psoriasis Foundation. He is founder and majority owner of www.DrScore.com and founder and part owner of Causa Research, a company dedicated to enhancing patients’ adherence to treatment. The remaining author states no conflict of interest.

## Declaration of Generative Artificial Intelligence (AI) or Large Language Models (LLMS)

The author(s) did not use AI/LLM in any part of the research process and/or manuscript preparation.
